# The Need for Antiviral Drugs for Pandemic Coronaviruses From a Global Health Perspective

**DOI:** 10.3389/fmed.2020.596587

**Published:** 2020-12-22

**Authors:** Angela Holly Villamagna, Sara J. Gore, James S. Lewis, J. Stone Doggett

**Affiliations:** ^1^Division of Infectious Diseases, Oregon Health & Science University School of Medicine, Portland, OR, United States; ^2^Department of Hospital and Specialty Medicine, VA Portland Healthcare System, Portland, OR, United States

**Keywords:** drug discovery, global health, viral pneumonia, antiviral drugs, outbreak preparedness, COVID-19, coronavirus, target product profile

## Abstract

Respiratory failure due to SARS-CoV-2 has caused widespread mortality, creating an urgent need for effective treatments and a long-term need for antivirals for future emergent coronaviruses. Pharmacotherapy for respiratory viruses has largely been unsuccessful with the exception of early treatment of influenza viruses, which shortens symptom duration and prevents infection in close contacts. Under the rapidly evolving circumstances of the COVID-19 pandemic, most clinical trials of experimental treatments in the United States have focused on later stages of the disease process. Worldwide, the clinical studies of the most impactful drugs, remdesivir and dexamethasone in ACTT-1, RECOVERY, and Solidarity, have studied hospitalized patients. Less than half of clinical trials in the U.S. have investigated oral agents, and the majority have taken place in hospitals at a disease stage where the viral load is already decreasing. The limited success of treatments for respiratory viruses and the viral dynamics of COVID-19 suggest that an antiviral therapy with the greatest impact against pandemic coronaviruses would be orally administered, well-tolerated, target a highly conserved viral protein or host-coronavirus interaction and could be used effectively throughout the world, including resource-poor settings. We examine the treatment of respiratory viral infections and current clinical trials for COVID-19 to provide a framework for effective antiviral therapy and prevention of future emergent coronaviruses and call attention to the need for continued preclinical drug discovery.

## Introduction

The emergence of SARS-CoV-2 has caused a devastating pandemic that has crippled healthcare systems, destroyed economies, and killed more than one million people. SARS-CoV-2 has eclipsed previous emergent coronaviruses in its global reach, and though the extent of the pandemic remains to be seen, models have predicted that significant transmission will occur through 2022 and resurgences will be possible through 2024 (1). Whether SARS-CoV-2 transmission persists or not, other emergent coronaviruses remain an ongoing threat to global health. Outbreaks of SARS-CoV, SARS-CoV-2, and MERS-CoV have demonstrated the high pathogenicity and mortality of zoonotic coronaviruses when they infect humans. By comparison, the SARS-CoV outbreak resulted in close to 8,000 cases with a 9.6% case-fatality rate and MERS-CoV has resulted in multiple clusters since 2012 with a case fatality rate of up to 40%, while the SARS-CoV-2 case fatality rate is estimated to be 2.3% (2, 3). Moreover, multiple zoonotic coronaviruses are capable of infecting humans and could potentially lead to future pandemics (4).

An effective antiviral that has broad activity against coronaviruses would decrease the impact of future emerging coronaviruses by preventing deaths and slowing viral transmission while public health measures are put into place and vaccines are developed. Large scale efforts are underway to find drugs that can be repurposed to treat COVID-19. Clinical trials and high throughput screens of repurposed drugs may reveal a safe and effective drug that coincidentally treats COVID-19; however, drugs that are discovered by this approach will likely need further structural optimization to increase antiviral efficacy against coronaviruses or decrease side effects. Clearly defining the essential characteristics of an effective anti-coronavirus treatment during these early stages is important to ensure that investments in drug development are allocated strategically, and that the search does not end prematurely or fail due to waning interest from public funding agencies and the pharmaceutical industry.

## Antiviral Drugs for Viral Lower Respiratory Tract Infection

Viral respiratory disease mortality is remarkably difficult to reduce with antiviral medications. For most respiratory viral diseases, antiviral treatment is limited to severe cases in vulnerable populations due to a lack of effective therapies. For example, the only antiviral therapy currently utilized for measles is ribavirin. Based on a single 2011 randomized trial of 100 children that showed ribavirin decreased duration of fever and symptoms (5) and a limited number of case series, ribavirin is utilized for people with measles who are profoundly immunocompromised or who have severe pneumonia, to unclear benefit. Limited drug development for more common respiratory viruses, such as adenovirus, is likely related to the low incidence of severe lower respiratory tract disease. Cidofovir is the only consistently utilized antiviral for severe adenovirus based on case series showing clinical improvement in hematopoietic stem cell transplant recipients with severe adenovirus disease (6, 7). However, cidofovir has not been studied in randomized controlled trials (RCT) and has a high rate of severe adverse effects. Similarly, though RSV is common, supportive measures rather than antivirals are the mainstay of treatment. Studies of ribavirin are contradictory and have not consistently shown clinical benefit in RSV lower respiratory tract infection (8). Like cidofovir for adenovirus, the evidence of benefit from ribavirin for RSV in the treatment of adult stem cell transplant recipients is limited to observational studies (9). Despite limited efficacy in treating RSV lower respiratory tract infection, prophylaxis using palivizumab, a monoclonal antibody that targets the RSV fusion glycoprotein, has been found to reduce the incidence of severe lower respiratory tract infection among children with chronic lung disease, congenital heart disease or a history of premature birth (10). The absence of effective treatment for clinically significant lower respiratory tract viral infection may reflect both the lack of resources devoted to drug discovery and the inherent limitations of antivirals for viral respiratory infections.

Antivirals often do not change outcomes because most respiratory viral infections are self-limited, viral replication is often waning at the time that symptoms develop and antivirals are administered too late. Moreover, severe disease manifestations, such as acute respiratory distress syndrome, are primarily driven by host-mediated inflammation rather than ongoing viral replication. The possible efficacy of antivirals in immunocompromised patients and as prophylaxis suggests that antivirals alter the progression of disease during active viral replication and tissue spread when viral replication is not already inhibited by the early host immune response. That said, the lack of antiviral efficacy against the above-mentioned infections may also be due to the limited intrinsic potency of the antivirals that were repurposed to treat these infections.

Influenza treatment is a notable exception, for which oral antivirals decrease symptoms, are well-tolerated, and are effective as prophylaxis. Neuraminidase inhibitors (NAIs), such as oseltamivir, have been mainstays of influenza treatment. Inhibition of viral neuraminidase prevents cleavage of host cell membrane glycoproteins and release of influenza virions. More recently, baloxavir marboxil, a cap-dependent endonuclease inhibitor, has also proven effective against influenza. The success of antivirals with varied mechanisms of action against the influenza viruses indicate that influenza does not have a unique Achilles' heel that results in susceptibility to antivirals. Accordingly, coronavirus inhibitors targeting essential stages of viral proliferation would be expected to decrease the severity of disease if administered early enough to reduce the viral burden.

Both preclinical animal studies and clinical studies have shown that influenza treatment with oseltamivir within 36 h of symptoms shortens the duration of symptoms compared to placebo (11–14). Given the incubation period of 24–48 h for influenza (15), these patients were likely treated 60–84 h post-infection. Observational studies highlight the real-world challenges of initiating influenza treatment within 36 h. The majority of patients present 72 h or more after developing symptoms (16, 17). The role for antivirals early in the disease is well-demonstrated but mortality benefit later in the disease course is less clear (18, 19). In addition to treatment, oseltamivir and baloxavir have demonstrated efficacy as prophylactic agents for household contacts of people with influenza (20, 21). Compared to the influenza viruses, the value of prophylaxis is greater for highly pathogenic coronaviruses due to the higher mortality, immunologically naïve population and the prolonged incubation period and transmission during the asymptomatic phase (22). An effective, well-tolerated, orally administered prophylactic drug could both prevent progression to severe disease and play a crucial role in limiting SARS-CoV-2 transmission alongside aggressive testing strategies.

## COVID-19 Treatment Efficacy In Clinical Trials

The understanding of SARS-CoV-2 viral dynamics is rapidly evolving, but two quantitative PCR studies showed the highest viral loads at or just after symptom onset, with a subsequent gradual decline (23, 24). These data suggest that the viral dynamics are similar to influenza viruses, in which viral load peaks on the day of symptom onset (15). This indicates that starting antiviral therapy as close to symptom onset as possible, or after a high-risk exposure, has the greatest chance to reduce the viral burden of disease and pathology. That said, antiviral treatment of COVID-19 at a median of 9 days of symptoms has led to more rapid resolution of symptoms in certain patients, indicating that window for antiviral intervention may be longer for COVID-19 than it is for influenza (25).

The timing of antiviral therapy initiation in clinical trials of COVID-19 therapies has varied widely but has generally been later in the disease course after patients are hospitalized. The median time from symptom onset to randomization has been as long as 30 days but, for the most part, studies have enrolled patients with a median time from illness onset to randomization of 9–13 days (10, 26–29).

The results of RCTs that have shown clinical benefit suggest that antivirals are less effective in advanced disease. The ACTT-1 remdesivir trial reported a median of 9 days from symptom onset to randomization (25). Whereas, there was no significant difference between groups that received remdesivir before or after 10 days of symptoms, patients receiving mechanical ventilation or extracorporeal membrane oxygenation did not benefit from remdesivir like patients with less severe disease. Subsequent results from the Solidarity trial did not show a clear benefit from remdesivir; however, the duration of symptoms prior to treatment was not reported (30). In RCTs that evaluated earlier time points, favipiravir plus interferon-α reported clinical improvement in subjects with symptoms ≤7 days, and in a randomized open-label trial of lopinavir-ritonavir, interferon beta-1b, and ribavirin compared to lopinavir-ritonavir alone, *post-hoc* subgroup analysis showed a shorter time to negative PCR and clinical improvement in subjects treated within 7 days of symptom onset, but no benefit if treated later (31, 32). Subsequent trials of interferon have shown mixed results (30, 33, 34). More recently, monoclonal antibodies targeting the SARS-CoV-2 spike protein are reported to possibly reduce hospitalizations when given within a median of 4 days of symptoms, whereas, monoclonal antibody therapy was not effective in hospitalized patients (35).

**Figure 1:**
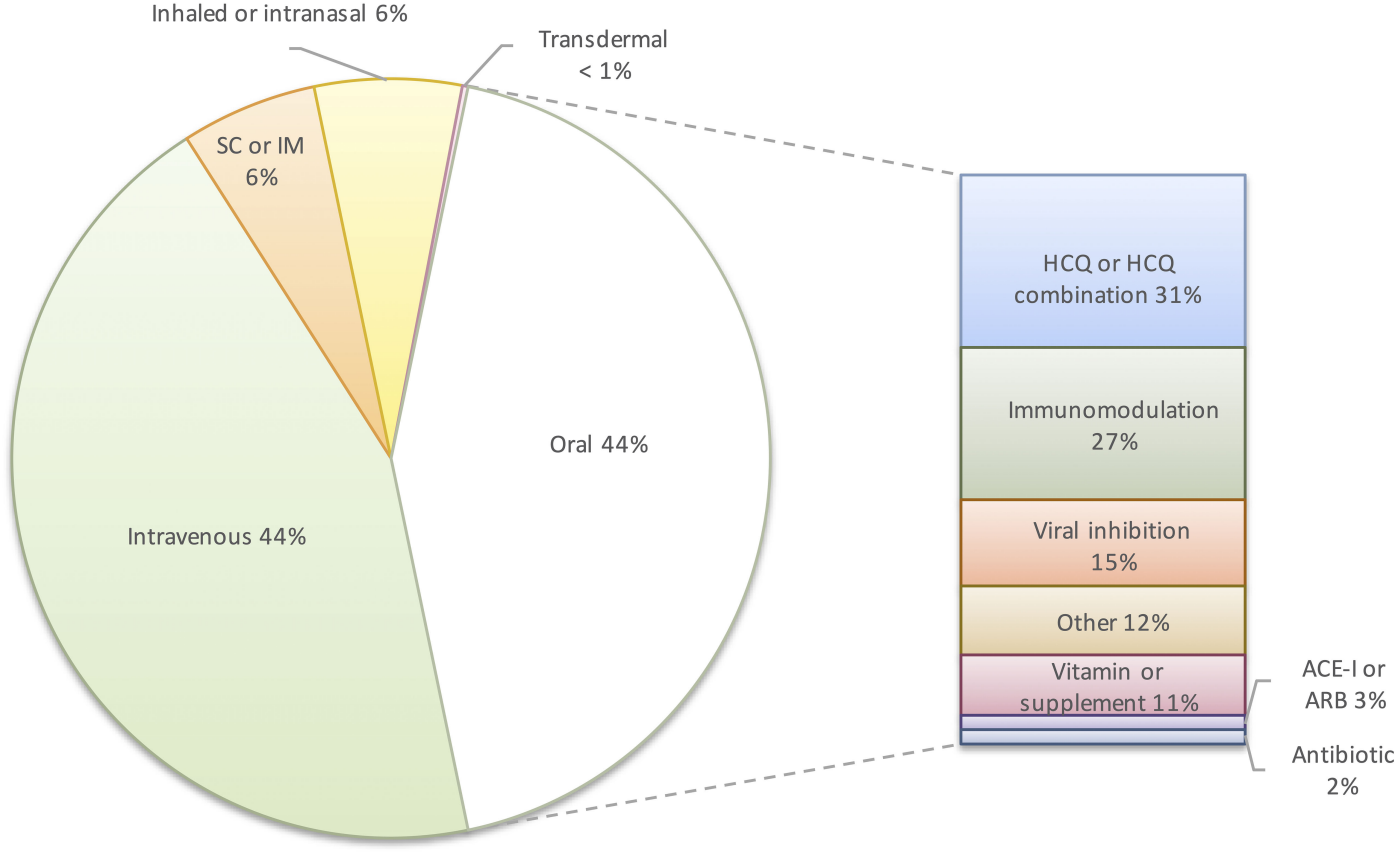
Distribution of route of administration and types of oral pharmacologic intervention in clinical trials for COVID-19 (*n* = 414). SC, subcutaneous; IM, intramuscular; HCQ, hydroxychloroquine; ACE-I, angiotensin converting enzyme inhibitor; ARB, angiotensin receptor blocker.

Conversely, evidence from clinical trials suggests treatment of COVID-19 with immunosuppressive therapies is effective after patients require supplemental oxygen and may be harmful if given too early. For example, while the dexamethasone arm of the RECOVERY trial showed a reduction in 28-day mortality in hospitalized patients with COVID-19 requiring supplemental oxygen or mechanical ventilation, there was no mortality benefit for patients not receiving supplemental oxygen or with <7 days of symptoms (36). This is notable given that 27% of oral drugs in clinical trials are immunomodulators (Figure 1). That said, fluvoxamine, which is presumed to modulate the host response to COVID-19 by interacting with the human sigma-1 receptor, was recently found to decrease clinical deterioration in a small trial (37). This finding, if replicated in larger trials, would support targeted immunomodulation early in disease. Immunosuppression has played a key role in reducing mortality in hospitalized COVID-19 patients; however, this approach would not limit the spread of infection as post- or pre-exposure prophylaxis and may not be applicable to future emerging coronaviruses.

## Current Clinical Trials of Drugs For Covid-19 In the United States

A comprehensive registry of COVID-19 trials listed 414 clinical trials of pharmacotherapeutic interventions in the United States as of November 1st, 2020 (38). Seventy-two trials studying vaccines, devices, oxygenation strategies, or other non-pharmacologic strategies were excluded. Of the 414 drug studies, 44% are of intravenous medications (Figure 1). The focus of clinical trials on hospitalized patients reflects efforts to treat severe disease. While this is an appropriate focus during a global health crisis, prior experience with respiratory viruses demonstrates marginal benefits from antivirals at this stage of disease. Ultimately, a drug administered early in infection to decrease viral transmission and to prevent progression from mild to severe disease will have the greatest impact. Only 44% of the current U.S. drug trials listed are studying oral therapies, and 31% of those studies involve hydroxychloroquine. Outpatient intravenous therapies such as remdesivir and the monoclonal antibodies, LY-CoV555 (bamlanivimab) and REGN-COV2, may limit disease progression in high-risk populations in resource-rich countries, but the cost, scale of production, and infrastructure required for intravenous administration prohibit their use as prophylaxis or treatment on a global scale (35). The prolonged time required to develop monoclonal antibodies for a novel virus also precludes them from being an initial response to an emerging pandemic virus.

Only 7% of clinical trials evaluating pharmacologic treatments for COVID-19 in the US are evaluating oral drugs with proposed antiviral mechanisms. Of the RCTs of outpatient oral treatment, 11 drugs have *in vitro* evidence of SARS-CoV-2 inhibition: AT-527, camostat mesylate, dipyridamole, ebselen, EIDD-2801, favipiravir, ivermectin, niclosamide, nitazoxanide, oleandrin, and toremifene (39–49). Of these drugs, only favipiravir has prior results from prospective clinical trials, which have had conflicting results (32, 50, 51). RCTs of favipiravir are ongoing in both the inpatient and outpatient setting. *In vitro* results for several of these drugs suggest that clinical efficacy is unlikely. Plasma concentrations achieved by current doses of ivermectin are far below the concentrations that are predicted to be required based on its inhibitory activity against SARS-CoV-2 in cell culture (41). The low selectivity index of toremifene of 2.8 indicates that *in vitro* activity may be related to host cell toxicity rather than efficacy, and toremifene would not be tolerated by patients at antiviral doses (46). Similarly, the therapeutic index of oleandrin is likely very narrow and the risk of overdose is high given that effective *in vitro* concentrations against SARS-CoV-2 overlap with plasma concentrations that have resulted in toxicity (49, 52, 53). Two oral prodrug compounds in particular, AT-527 and EIDD-2801, have shown promising broad active against multiple coronaviruses. EIDD-2801, a ribonucleoside analog, inhibits 50% of SARS-CoV-2 replication in cell culture at concentrations <0.1 μM, was active against SARS-CoV, MERS and several bat coronavirus strains, and reduced lung viral loads and improved pulmonary function in a mouse model of SARS-CoV and MERS (40). AT-527, a guanosine nucleotide analog previously studied in patients with hepatitis C, inhibited 90% of SARS-CoV-2 replication at a concentrations close to 0.5 μM, and was active against SARS-CoV and human coronaviruses, HCoV-229E and HCoV-OC43 (47). While encouraging that a few drugs in clinical trials could be repurposed to treat COVID-19 and emerging coronaviruses, this small number reveals a large unmet need for preclinical coronavirus drug development.

## A Target Profile for A Global Anti-Coronavirus Drug

Target product profiles are often constructed by industry, regulatory agencies, or public health organizations to strategically identify attributes required for drugs to meet essential needs. In the case of highly pathogenic coronaviruses, a target profile serves to define the minimal targets that should be met before drug discovery efforts cease rather than exclude drugs that currently offer incremental improvements (Table 1). After a successful COVID-19 vaccine is in widespread use, the economic incentives to discover drugs to treat COVID-19 and to prevent future coronavirus pandemics will be diminished. Defining benchmarks now will help set goals for drugs that could be stock-piled or ready for production and clinical testing in the event of another novel coronavirus outbreak and a consensus around preclinical development that will likely rely on funding from public agencies.

**Table 1:** Suggested drug profile.

**Parameter to be demonstrated**	**Minimum essential**	**Ideal**
Indication for use	• Post-exposure prophylaxis in close contacts of COVID-19 infected patients • Symptomatic with influenza-like illness for ≤72 h	• Post-exposure prophylaxis in close contacts of COVID-19 infected patients • Symptomatic with influenza-like illness for ≤96 h • Hospitalized patients with an oxygen saturation ≤94%
Target population	• Adults, including the elderly • Children • Pregnant women	• Adults, including the elderly • Children • Pregnant women
Formulation and route	• Oral	• Oral and intravenous, intramuscular or rectal
Clinical efficacy	• Decreased transmission in close contacts ≤50% of the naturally occurring rate • Decreased risk of hospitalization when administered within 72 h of symptom onset • Decreased risk of death ≥10% compared to no treatment in patients with confirmed infection when administered within 72 h of symptom onset	• Decreased transmission in close contacts ≤80% of naturally occurring rate • Decreased risk of hospitalization when administered within 96 h of symptom onset • Decreased risk of death ≥50% compared to no treatment in patients with confirmed infection when administered within 72 h of symptom onset, ≥20% at 96 h • Decreased risk of death ≥10% in patients with an oxygen saturation ≤94%
Susceptibility to resistance	• No immediate high-level resistance due to single point mutation after serial passage in culture without profound decrease in fitness	• No significant resistance after serial passage in culture without profound decrease in fitness • Drug target that not highly mutable or placed under selective pressure by drug
Spectrum of activity	• Active against betacoronaviruses	• Active against all alpha and betacoronaviruses and pandemic influenza viruses
Drug-drug interactions	• No unmanageable risk accounting for poly-pharmacy in elderly populations and critically ill patients.	• No identified risk accounting for poly-pharmacy in elderly populations and critically ill patients.
Safety and tolerability	• Few and manageable adverse events • No severe adverse events • No monitoring required	• Low incidence of mild adverse events • No severe adverse events • No monitoring required
Stability	• Stable for 3 years under controlled storage conditions • Stable for 6 months at 30 ± 2°C and 65 ± 5% relative humidity	• Stable for 5 years under controlled storage conditions • Stable for 12 months at 30 ± 2°C and 65 ± 5% relative humidity
Cost of production	• Amenable to rapid large-scale synthesis and global distribution	• Amenable to rapid large-scale synthesis and global distribution
Dosing regimen	• Three times daily	• Once daily

*Recommended characteristics of a drug for SARS-CoV-2 and future potential pandemic coronaviruses*.

The target population for a coronavirus antiviral should be as broad as possible and include children and pregnant women. Mortality due to SARS-CoV-2 is increased in older adults, but young adults and children have served to spread infection, and future coronaviruses may have higher mortality in younger populations. An oral formulation is necessary for a drug to be available on a global scale and in infrastructure-limited regions. Given that zoonotic coronaviruses are globally distributed, the next pandemic could emerge in a resource-poor setting. Ideally, an additional parenteral or rectal formulation would allow for treatment of patients who are too ill to take oral medications.

A key part of defining a desired antiviral profile is setting targets for efficacy of treatment and prophylaxis. Given the limited success of treating respiratory viral infections, the goal of a 10% reduction in mortality when given within 72 h is ambitious; however, the trend toward improved mortality with remdesivir in hospitalized patients and the possible protective effect of anti-SARS-CoV-2 monoclonal antibodies given with a median of 4 days of symptoms suggest that this target is possible for COVID-19 (25, 35). In considering efficacy as prophylaxis, high SARS-CoV-2 household transmission rates of up to 53% suggest that partial efficacy would provide significant benefit (54). Decreased transmission rates to ≤50% of the natural transmission rates is a modest goal compared to influenza prophylaxis with NAIs or baloxavir, but would have a tremendous impact given the high transmission rates of SARS-CoV-2 in a non-immune population when there is no vaccine (20, 21).

Safety, tolerability and a lack of drug-drug interactions are an essential quality of a broadly used drug. Based on experience with the COVID-19 pandemic, the more than 3 months that were required to create and distribute an accurate test for SARS-CoV-2 necessitated a symptom-based approach to identifying and managing cases. A symptom-based approach to treatment and prophylaxis of an influenza-like-illness would result in many more people being treated than are infected. Moreover, the higher incidence of severe disease in older populations underscores the increased risk of side effects and polypharmacy and the importance of limiting drug-drug interactions.

Each viral disease is unique; however, the early days of the COVID-19 pandemic and the 2009 H1N1 influenza pandemic revealed obstacles that should be anticipated. The cost and scalability of production as well as the capacity to stockpile drug is equally important. Consequently, a drug must have long-term stability under heat and humidity, not require a cold chain, and if stockpiled as bulk powder, such as was the case with oseltamivir for H1N1, the capacity to rapidly reconstitute and distribute the drug must be in place (55). Finally, a drug that targets conserved coronavirus proteins or host-pathogen interactions and is broadly active against identified human and bat coronaviruses will have the greatest chance of being active against emerging coronaviruses. Creating a desired drug profile for an evolving pandemic or an anticipated coronavirus pandemic is challenging. In fact, the degree of impact of oseltamivir on the H1N1 pandemic remains a subject of debate (56). That said, we have suggested long term aims in hopes that drug development efforts for the next coronavirus pandemic will not end prematurely with a drug that only benefits specific populations in resource-rich countries.

## Conclusion

Drug discovery efforts for respiratory viral illnesses have resulted in few effective treatments. For many of these illnesses, drug development is not a matter of urgency given the relatively rare occurrence of severe pneumonia; however, the significant global mortality of lower respiratory tract infections from RSV in children and influenza reveals an unmet need for therapeutic interventions and the challenges of developing a respiratory antiviral drug that prevents severe disease. The mortality and societal costs of the highly pathogenic coronaviruses, SARS-CoV, MERS, and SARS-CoV-2 clearly show the immeasurable value of a drug to prevent the spread of a pathogenic coronavirus or prevent clinical progression to severe disease. Based on similarity in viral kinetics between influenza and SARS-CoV-2, examples of effective treatments for influenza, and preliminary evidence from COVID-19 clinical studies, medicines that can be administered early after symptom onset or as prophylaxis should be a primary target of coronavirus drug development. To accomplish this aim, drugs should have the standard characteristics of being well-tolerated, limited drug interactions, adequate tissue concentrations and a high degree of potency. In addition to standard characteristics, a drug to combat a global pandemic should be suitable for a range of global conditions and circumstances. Most importantly, the drug should target a viral protein or host cell pathway that is highly conserved among coronaviruses. The small number of repurposed drugs that currently meet these criteria indicate the need for robust preclinical drug discovery.

Alongside the first wave of clinical trials aimed at exploring off-the-shelf COVID-19 drugs, a parallel effort has searched for hits using phenotypic high throughput drug screens, *in silico* modeling of small molecule inhibitors with SARS-CoV-2 proteins and identification of host cell drug targets that are needed for viral proliferation (46, 57–59). These preclinical efforts have identified compounds that have low nanomolar IC_50_s against SARS-CoV-2 (57, 60). Drugs and preclinical compounds identified up to this point may have limited impact on COVID-19 prior to widespread immunization, but they identify mechanisms and pharmacophores that serve as starting points for continued drug development. A deep pipeline of preclinical coronavirus drug candidates will be required to prepare for the next pandemic. Establishing long term research benchmarks to discover drugs with broad-spectrum activity against coronaviruses that will stop the next pandemic will be well worth the investment after the traditional financial incentives for drug development fade.

## Data Availability Statement

Publicly available datasets were analyzed in this study. This data can be found at: https://www.covid-trials.org.

## Author Contributions

AV and SG contributed equally in conceiving and writing the manuscript. JL contributed expertise and edited the manuscript. JD contributed to the conception, writing, and editing of the manuscript. All authors contributed to the article and approved the submitted version.

## Conflict of Interest

The authors declare that the research was conducted in the absence of any commercial or financial relationships that could be construed as a potential conflict of interest.
